# TRAIL signals through the ubiquitin ligase MID1 to promote pulmonary fibrosis

**DOI:** 10.1186/s12890-019-0786-x

**Published:** 2019-02-07

**Authors:** Adam M. Collison, Junyao Li, Ana Pereira de Siqueira, Xuejiao Lv, Hamish D. Toop, Jonathan C. Morris, Malcolm R. Starkey, Philip M. Hansbro, Jie Zhang, Joerg Mattes

**Affiliations:** 1 Experimental and Translational Respiratory Medicine Group, Level 2 East, Hunter Medical Research Institute, School of Medicine and Public Health, Faculty of Health, University of Newcastle, Callaghan, NSW 2308 Australia; 2 Priority Research Centre GrowUpWell, The University of Newcastle and Hunter Medical Research Institute, Newcastle, Australia; 3 Department of Respiratory and Critical Care Medicine, Second Affiliated Hospital of Jilin University, Changchun, Jilin, 130041 People’s Republic of China; 4 School of Chemistry, University of New South Wales, Sydney, New South Wales Australia; 5 Priority Research Centre for Healthy Lungs, The University of Newcastle and Hunter Medical Research Institute, Newcastle, Australia; 6 Paediatric Respiratory & Sleep Medicine Department, Newcastle Children’s Hospital, Kaleidoscope, Newcastle, Australia

**Keywords:** TRAIL, MID1, PP2A, Fibrosis, E3 ubiquitin ligase

## Abstract

**Background:**

Tumour necrosis factor-related apoptosis-inducing ligand (TRAIL) has previously been demonstrated to play a pro-inflammatory role in allergic airways disease and COPD through the upregulation of the E3 ubiquitin ligase MID1 and the subsequent deactivation of protein phosphatase 2A (PP2A).

**Methods:**

Biopsies were taken from eight IPF patients presenting to the Second Affiliated Hospital of Jilin University, China between January 2013 and February 2014 with control samples obtained from resected lung cancers. Serum TRAIL, MID1 protein and PP2A activity in biopsies, and patients’ lung function were measured. Wild type and TRAIL deficient *Tnfsf10*^*−/−*^ BALB/c mice were administered bleomycin to induce fibrosis and some groups were treated with the FTY720 analogue AAL(s) to activate PP2A. Mouse fibroblasts were treated with recombinant TRAIL and fibrotic responses were assessed.

**Results:**

TRAIL in serum and MID1 protein levels in biopsies from IPF patients were increased compared to controls. MID1 levels were inversely associated while PP2A activity levels correlated with DLco. *Tnfsf10*^*−/−*^ and mice treated with the PP2A activator AAL(s) were largely protected against bleomycin-induced reductions in lung function and fibrotic changes. Addition of recombinant TRAIL to mouse fibroblasts in-vitro increased collagen production which was reversed by PP2A activation with AAL(s).

**Conclusion:**

TRAIL signalling through MID1 deactivates PP2A and promotes fibrosis with corresponding lung function decline. This may provide novel therapeutic targets for IPF.

## Introduction

Idiopathic pulmonary fibrosis (IPF) is the most common form of the idiopathic interstitial lung disorders accounting for 50–60% of cases [1]. There is marked heterogeneity in the histopathological findings, radiological profiles, treatment responsiveness and the rate of disease progression across individuals diagnosed with IPF. This is thought to be due to the complexity of the interplay between environmental and endogenous factors and the multiplicity of co-activated pathogenic pathways that have been demonstrated to underpin this disease [2].

IPF consistently clinically manifests as chronic and progressive exertional dyspnoea and dry cough of gradual onset as a result of restrictive ventilation impairment and respiratory failure [1]. Current medical interventions for IPF are either supportive therapy (oxygen therapy) or lung transplantation, and few effective pharmacological therapies are available for its treatment. IPF has a poor prognosis with a median survival of 3 years and a 5 year survival rate of 45% [3]. The traditional concept of IPF pathogenesis was that the initial event is an inflammatory process characterised by infiltration of inflammatory cells such as macrophages, lymphocytes and neutrophils in the alveoli (alveolitis). Inflammation was believed to lead to recruitment of fibroblasts and myofibroblasts and finally to collagen formation and irreversible fibrosis. However, clinical trials of drugs inhibiting an underpinning inflammatory process such as Etanercept, a TNF-α antagonist, have consistently failed to show any efficacy in slowing disease progression [4]. In contrast, antifibrotic drugs such as nintedanib and pirfenidone have been demonstrated to slow disease progression and have recently been approved for use in treatment of IPF in the USA and Europe [5, 6]. Thus, the current consensus is that IPF is a primary epithelial and fibroblastic disease, and pro-inflammatory and inflammatory mediators are involved in the epithelial-dependent fibrotic pathway.

Tumour necrosis factor-related apoptosis-inducing ligand (TRAIL) is a member of the tumour necrosis factor family that is principally expressed by airway epithelial cells [7, 8], and leukocytes including monocytes [9], macrophages [10], lymphocytes [11] and neutrophils [12]. It is best described for its role in inducing apoptosis in a variety of transformed cells [13–15]; however, we have recently described the fibrogenic role played by TRAIL signalling in mouse models of allergic and neonatal infection-induced airways disease and in eosinophilic esophagitis [16–18]. We have also shown that TRAIL induces the E3 ubiquitin ligase midline-1 (Mid1) to inhibit the activation status of the protein phosphatase (PP)2A by interacting with the α4 regulatory subunit that is required for the ubiquitin-specific modification and proteasome-mediated degradation of its catalytic subunit PP2Ac [19–21]. PP2A is the most abundant cellular protein phosphatase. Reduced activity due to Mid1 upregulation resulted in hyperphosphylation of mitogen-activated protein kinases (MAPKs) and inhibitor of κBα (IκBα) protein, thereby promoting p38 MAPK, c-Jun N-terminal kinase (JNK) and nuclear factor-κB (NF-κB) activity [22–26].

Here we investigate lung tissue derived from IPF patients for the pro-inflammatory fibrogenic upregulation of MID1 and PP2A. We also investigate the role of TRAIL-Mid1-PP2A signalling in the rodent fulminant-induced bleomycin model of pulmonary fibrosis and explore its relevance to collagen production by lung fibroblasts - thought to underpin much of the disease phenotype in human disease.

## Methods

Lung biopsies were collected from eight IPF patients from the Department of Respiratory and Critical Care Medicine, the Second Affiliated Hospital of Jilin University between January 2013 and February 2014. A clinical diagnosis of IPF was identified according to histological confirmation of usual interstitial pneumonia (UIP) or the presence of a UIP pattern on high-resolution computed tomography (HRCT). A total of 21 candidates without known ILD (eg. environmental factors, occupational exposure, connective tissue diseases, drug injury) underwent blood tests for screening of connective tissues diseases (including; ANCA, ANA, rheumatic blood test, complement C3,C4) and upon negative results underwent HRCT. Six patients were categorized as definite UIP and 4 as not UIP from the HRCT and did not undergo transbronchoscopic biopsy. The remaining 11 patients classified as possible UIP according to the ATS/ERS/JRS/ALAT statement criteria underwent transbronchosopic biopsy of which eight had histologically confirmed UIP. The ATS/ERS/JRS/ALAT guidelines state that “the sensitivity and specificity of this approach (transbronchosopic biopsy) for the diagnosis for UIP pattern is unknown” and therefore not always suitable for the diagnosis of IPF. However, in each of the eight cases here the diagnosis of IPF was made on the basis of all features present (clinical parameters, CT images, histology). Importantly, control tissues were from tissues distal to resected lung cancer in patients with no clinical evidence of IPF or other fibrotic lung disease. Written informed consent was obtained from individual subjects, and all the studies were approved by the Medical Ethics Committee of the Second Affiliated Hospital of Jilin University, Changchun, Jilin, P.R. China.

### Mice

Wild-type (WT) and TRAIL deficient (*Tnfsf10*^−/−^) male BALB/c mice (6–14 weeks-old) were obtained from the Australian Bioresources facility (Moss Vale, Australia). Animals were housed with ad libitum access to food and water with a 12-h light-and-dark cycle.

### Bleomycin-induced pulmonary fibrosis

Mice were anaesthetised with Alfaxan by tail vein injection, then 50 μl solution containing bleomycin (2 mg/kg, Sigma-Aldrich, USA) was intratrachealy administered. Control mice were administered saline instead. All procedures were performed in a sterile environment [27]. In order to activate PP2A, mice received the nonphosphorylatable FTY720 analog, (S)-2-amino-4-(4-heptyloxyphenyl)-2-methylbutan-1-ol (AAL(s)) intranasaly starting at day − 1 and daily throughout the model with bleomycin administration at day 0. Mice were sacrificed 1, 4, 8, or 21 days after bleomycin instillation via pentobarbitone overdose, and one lung was taken for histology and one was homogenised for protein quantification.

### Lung capacity

Vital capacity was assessed under Ketamine and Xylazine induced anaesthesia, as the volume of air that entered the lungs when the airway pressure was increased from 2 to 30 cmH_2_O by the ventilator (PVs-P Flexivent manoeuvre). Compliance was calculated as the measured change in volume divided by this applied pressure change. Three inflations were performed and averaged per mouse [27, 28].

### Histological preparations

Lungs were collected in 10% neutral buffered formalin solution for 24 h before being transferred to 70% ethanol. Tissue was then embedded in paraffin and stained with either hematoxylin and eosin (eosinophils), periodic acid Schiff (PAS positive mucus producing epithelial cells), Masson’s Trichrome (Collagen), toluidine blue (mast cells) or processed further for immunohistochemistry. Lungs across experimental groups were all processed as a batch for either histologic staining or the immunostaining protocol.

### Collagen area enumeration

The area of peribronchial trichrome staining in a paraffin-embedded lung was outlined and quantified using a light microscope (Olympus) attached to an image-analysis system (Image-Pro Plus 6; Media Cybernetics, Maryland, USA). Results are expressed as the area of trichrome staining per micrometre length of basement membrane of major airways. At least 10 counts were made from each mouse as previously described [17, 29].

### Terminal dUTP nick end labelling (TUNEL) assay

Longitudinal sections of the left single-lobe lung were stained with TUNEL assay kits (Promega, Sydney, Australia) according to the manufacturer’s instructions. Apoptosis in lung parenchyma was assessed by enumerating the numbers of TUNEL positive and negative cells in 20 randomised, 100mm^2^ fields by light microscopy [28].

### Quantitative RT-PCR

We performed quantitative RT-PCR with SYBR Green (Invitrogen, Sydney, Australia) on a Realcycler (Eppendorf, Sydney, Australia) with the following cycling conditions: 2 min at 50 °C followed by 2 min at 95 °C. Amplification was recorded during 40 cycles of 15 s at 95 °C followed by 45 s at 60 °C. We quantified mRNA copy number using cDNA standards for all genes of interest. We normalised expression to the housekeeping gene Hprt with all primer sequences previously described [22].

### Elisa

Concentrations of human MID1 (Cusabio, USA) and PP2A activity (R&D systems, USA) in homogenized lung biopsies and TRAIL (R&D systems, USA) in serum were determined using a sandwich ELISA according to the manufacturer’s instructions [30, 31].

### Primary mouse fibroblast culture

Lung tissue was collected under sterile conditions and cut into approximately 1 × 1 mm tissue fragments. They were washed three times with PBS and then spread on the bottom of 6-well plates in complete DMEM with 10% (vol/vol) FCS. The media was changed every 2–3 days. Cell monolayers formed around tissue blocks in 1–2 weeks. Tissue fragments were removed using sterile forceps if more than 60% of the plate was covered by attached fibroblasts. When fibroblasts reached > 90% confluence, 0.25% trypsin was used to lift the cells, which were then removed in two culture flasks for passage (1:2). Fibroblasts were passaged 3 times with this method. We used passage 4 and cultured them until 80% confluence, serum-starved them for 24 h and incubated them with recombinant (r)TRAIL (0 ng/ml-100 ng/ml) for 24 h in serum-free DMEM.

### Statistical analysis

Statistical significance was analysed using Student’s t-test or Mann-Whitney test as appropriate. All graphed data are expressed as mean ± SEM. Correlation analysis was done using a Spearman test. All data was analysed using GraphPad Prism 7 (La Jolla, CA).

## Results

### IPF diagnosis

**Fig. 1:**
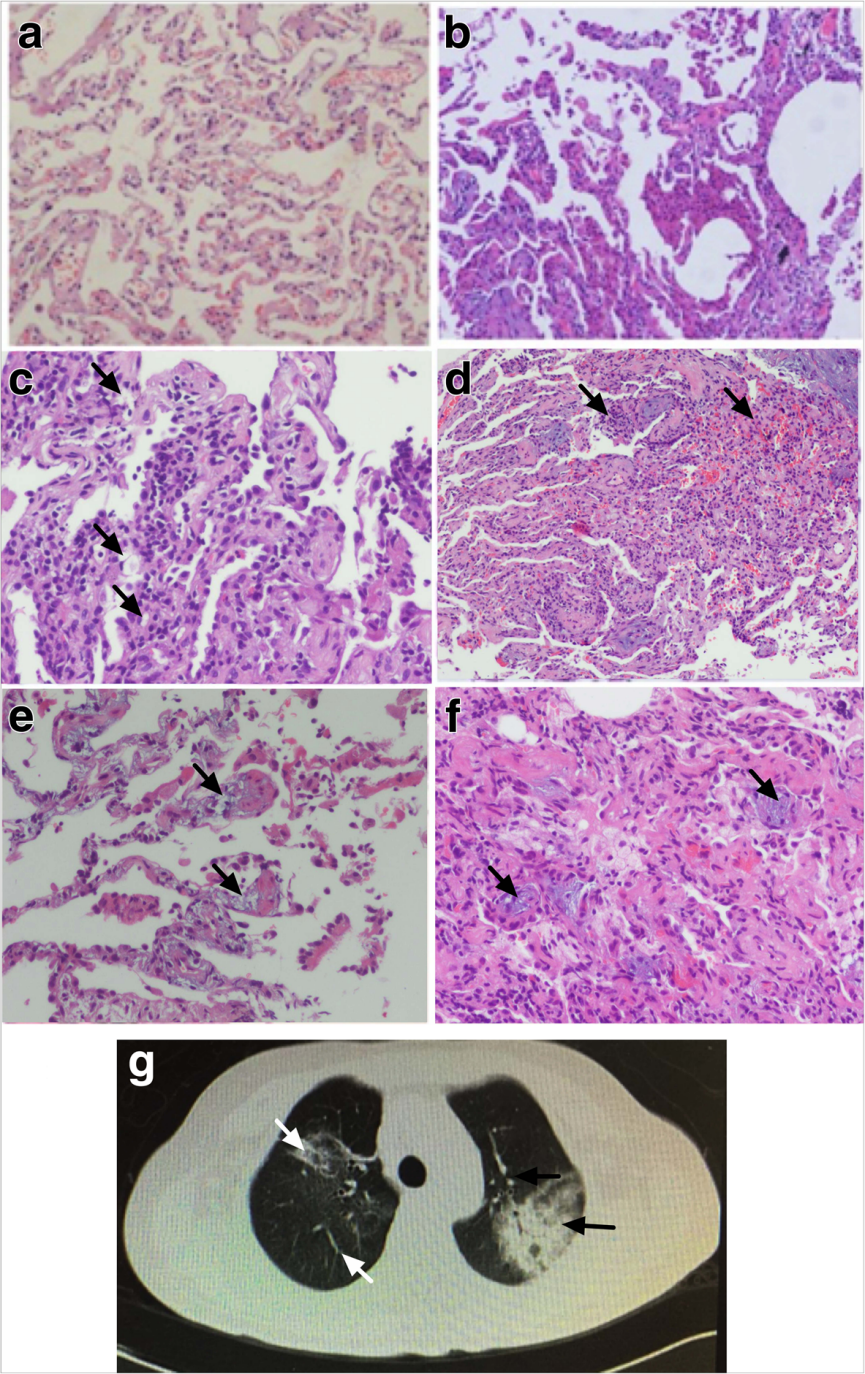
Lung and alveolar structure is disrupted, and inflammatory responses occur in IPF. Representative images of haematoxylin and eosin stained transbronchial biopsy sections at 200x magnification with arrows highlighting described features: Normal lung architecture from control patients (**a**), distortion of the normal lung architecture and obliteration of alveolar architecture consistent with IPF (**b**), hyperplasic pneumocytes (**c**), inflammatory infiltrates (**d**), and fibroblastic proliferation (**e**, **f**). Representative high-sresolution computerised-tomography image showing areas of air-space consolidation, ground glass opacities, small nodular opacities and bronchial wall thickening and dilatation (**g**)

Patients with a diagnosis of IPF as expected had a reduced percentage predicted carbon monoxide diffusing capacity (% pred DLco) and forced vital capacity (% pred FVC) (Table 1). Controls had normal DLCO and FVC. Transbronchial biopsy sections for IPF patients had distorted lung architecture and obliterated alveolar structure with hyperplastic pneumocytes, inflammation and fibroblast proliferation (Fig. 1).

**Table 1:** Participant demographics for the control and IPF populations included in the study

	Control	IPF	*p* value
Gender (male/ total)	5/8	5/8	
Age (min-max)	56 (45–62)	65 (50–83)	0.08
dLCo (25th–75th)	92.27 (89.2–96.53)	43.85 (28.73–53.73)	< 0.001
FVC (25th – 75th)	79.85 (78.5–80.8)	44.2 (28.75–51.5)	< 0.001
Smokers	2/8	3/8

### TRAIL in serum and MID1 protein in biopsies are increased in IPF

**Fig. 2:**
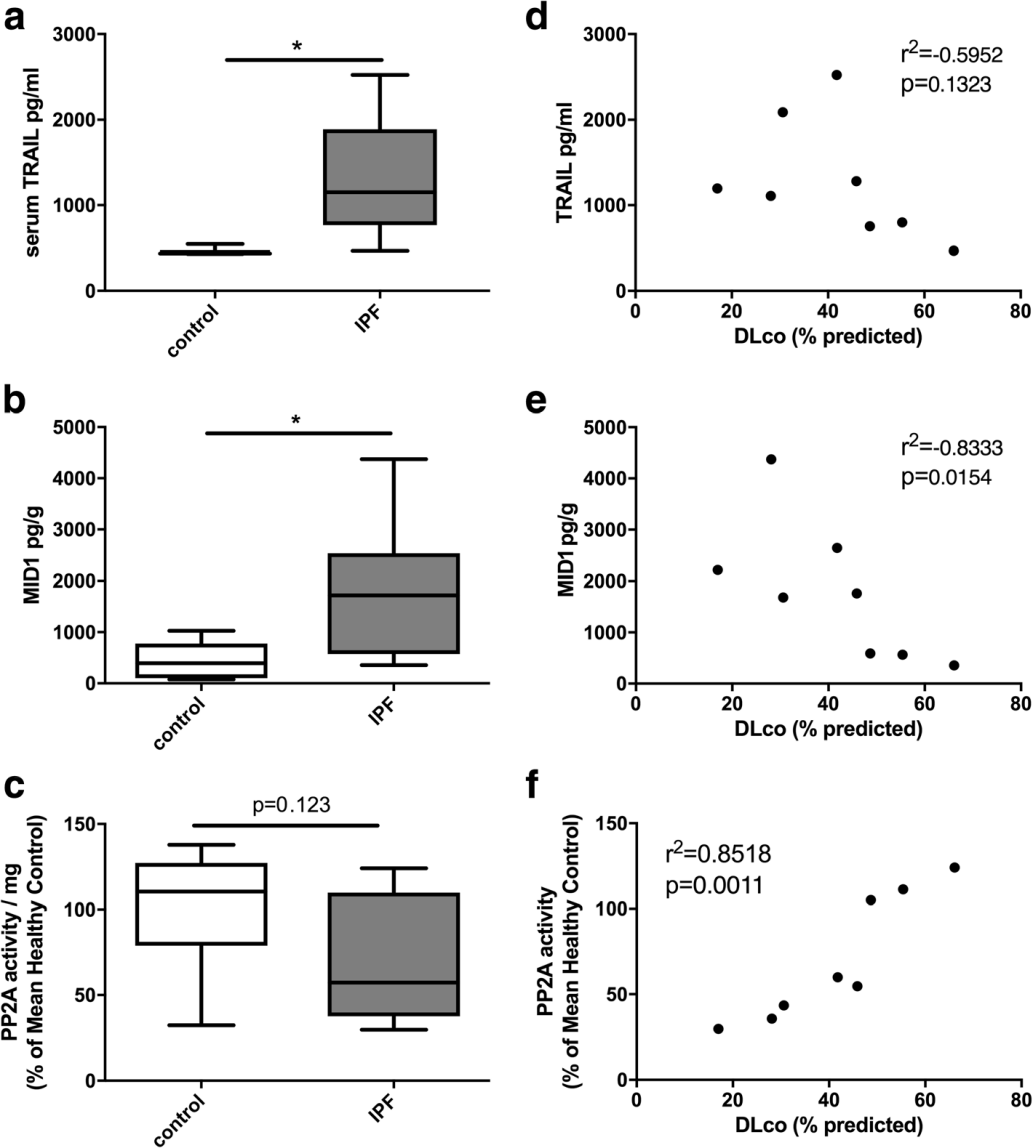
Serum from IPF patients had elevated levels of TRAIL (**a**) and lung biopsies elevated MID1 (**b**) protein, which corresponded with a trend towards decreased activity of PP2A (**c**) when compared to those from control subjects. The percentage predicted diffusing lung capacity for carbon monoxide (DLco) did not have a strong inverse association with serum TRAIL (**d**) but was inversely associated with MID1 protein (**e**) and correlated with the levels of PP2A activity (**f**) in lung biopsies from IPF patients. *n* = 8, * *p* < 0.05

We assessed protein levels of TRAIL in the serum and MID1 and phosphatase activity of PP2A in homogenised lung specimens compared to controls. We found that TRAIL and MID1 protein levels were significantly upregulated in IPF patients (Fig. 2a, b). As expected, IPF patients had a reduced percentage predicted carbon monoxide diffusing capacity (% pred DLco) (Table 1). Notably MID1 protein and PP2A activity levels in lung biopsies from IPF patients correlated with DLco (Fig. 2e, f).

### Elevated Mid1 and reduced PP2A activity occurs in bleomycin-induced pulmonary fibrosis, and the absence of TRAIL or activation of PP2A reverses these effects and improve lung function

**Fig. 3:**
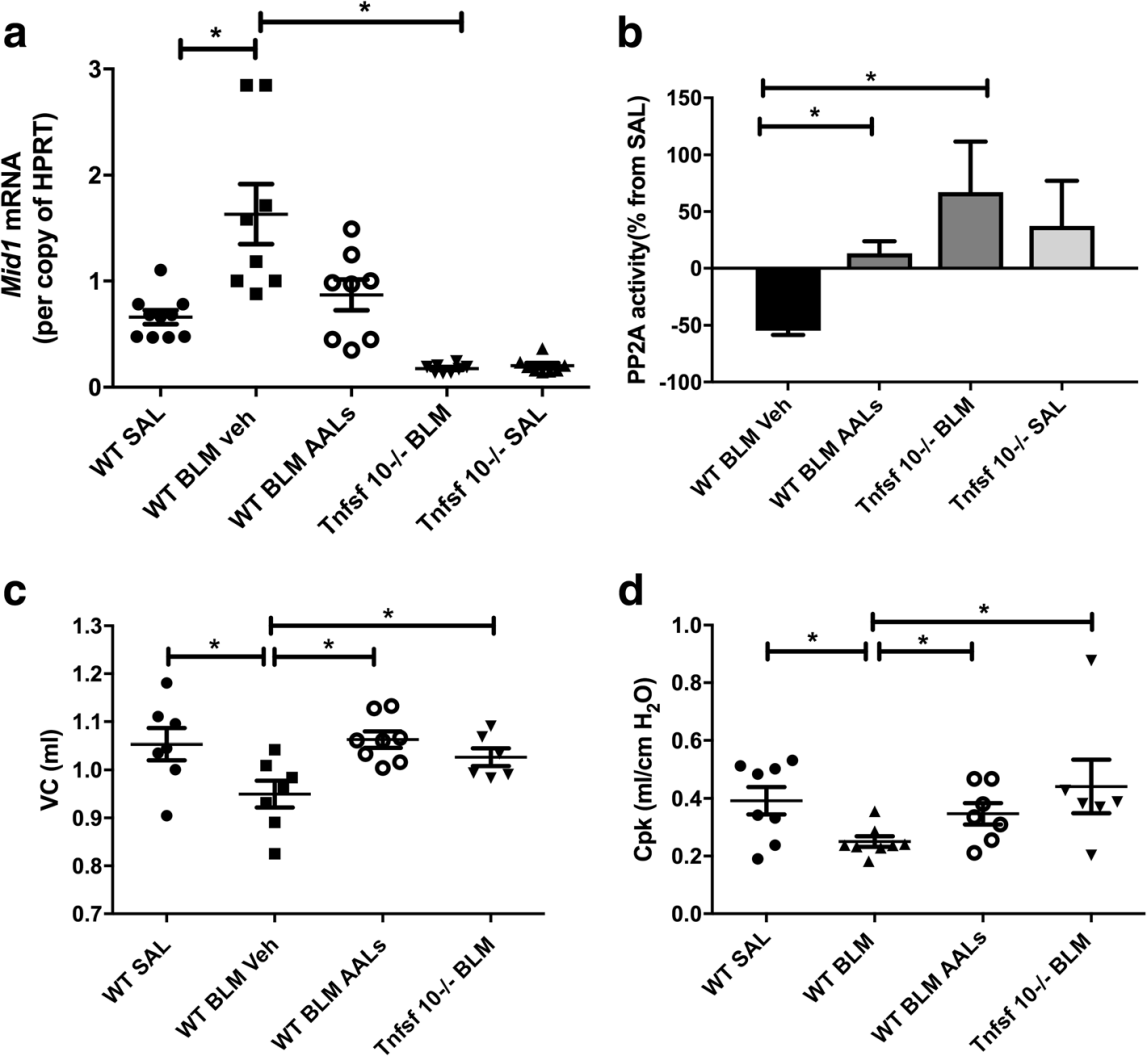
Wild-type mice treated with the PP2A activator AAL(s) and *Tnfsf10*^*−/−*^ mice were protected from increases in Mid1 mRNA, and the loss of PP2A activity and lung function induced by bleomycin. Mid-1 mRNA was downregulated in AAL(s) treated WT mice and *Tnfsf10*^*−/−*^ mice exposed to bleomycin (**a**) while PP2A activity was increased (**b**). The vital capacity (VC) and pressure at the peak of compliance (Cpk) were both decreased by bleomycin 21 days post exposure but *Tnfsf10*^*−/−*^ mice or those treated with AAL(s) were protected (**c**-**d**). *n* = 5–8, * *p* < 0.05

We then assessed a mouse model of lung fibrosis induced by bleomycin. In support of the human data WT BALB/c mice with bleomycin-induced lung fibrosis displayed a marked upregulation of Mid1 and a downregulation of PP2A activity in their lungs (Fig. 3a, b). Furthermore, WT mice treated with the synthetic PP2A activator AAL(s) and *Tnfsf10*^−/−^ mice were protected from the upregulation of Mid1 and the deactivation of PP2A in response to bleomycin exposure (Fig. 3a, b). WT mice treated with AAL(s) and *Tnfsf10*^−/−^ mice were also protected from the bleomycin-induced decline in lung function as shown by an unaffected vital capacity (VC) and peak compliance in response to bleomycin exposure (Fig. 3c, d).

### Pro-fibrotic factor mRNA levels are increased in bleomycin-induced pulmonary fibrosis in mice, and the absence of TRAIL or activation of PP2A reverses these effects

**Fig. 4:**
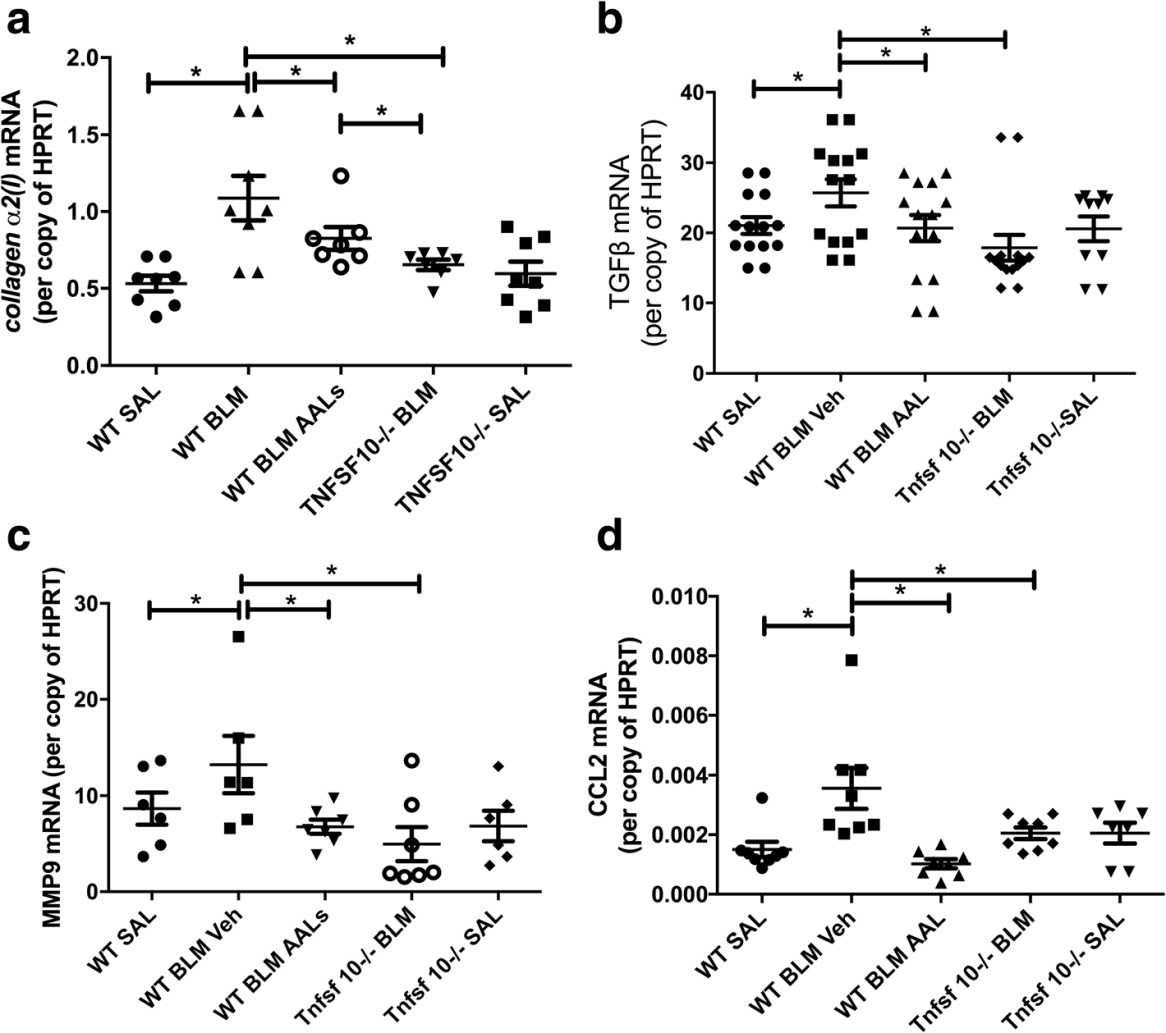
Wild type mice treated with AAL(s) and *Tnfsf10*^−/−^ mice had reduced expression of collagen-α2(I) (**a**), TGFβ (**b**), mmp9 (**c**) and ccl2 (**d**) in their lungs 21 days after bleomycin exposure. *n* = 7–12, * *p* < 0.05

We next investigated the expression of pro-fibrotic factors and found that bleomycin-exposed WT mice had elevated levels of collagen-α2(I), transforming growth factor β1 (TGFβ), matrix metalloproteinase (MMP) 9 and C-C motif chemokine ligand (CCL) 2 mRNA. AAL(s) treated WT mice and *Tnfsf10*^*−/−*^ mice were protected from the increases in these pro-fibrotic mediators (Fig. 4).

### Collagen deposition and apoptosis levels are increased in bleomycin-induced pulmonary fibrosis in mice, and the absence of TRAIL and activation of PP2A reverses these effects

**Fig. 5:**
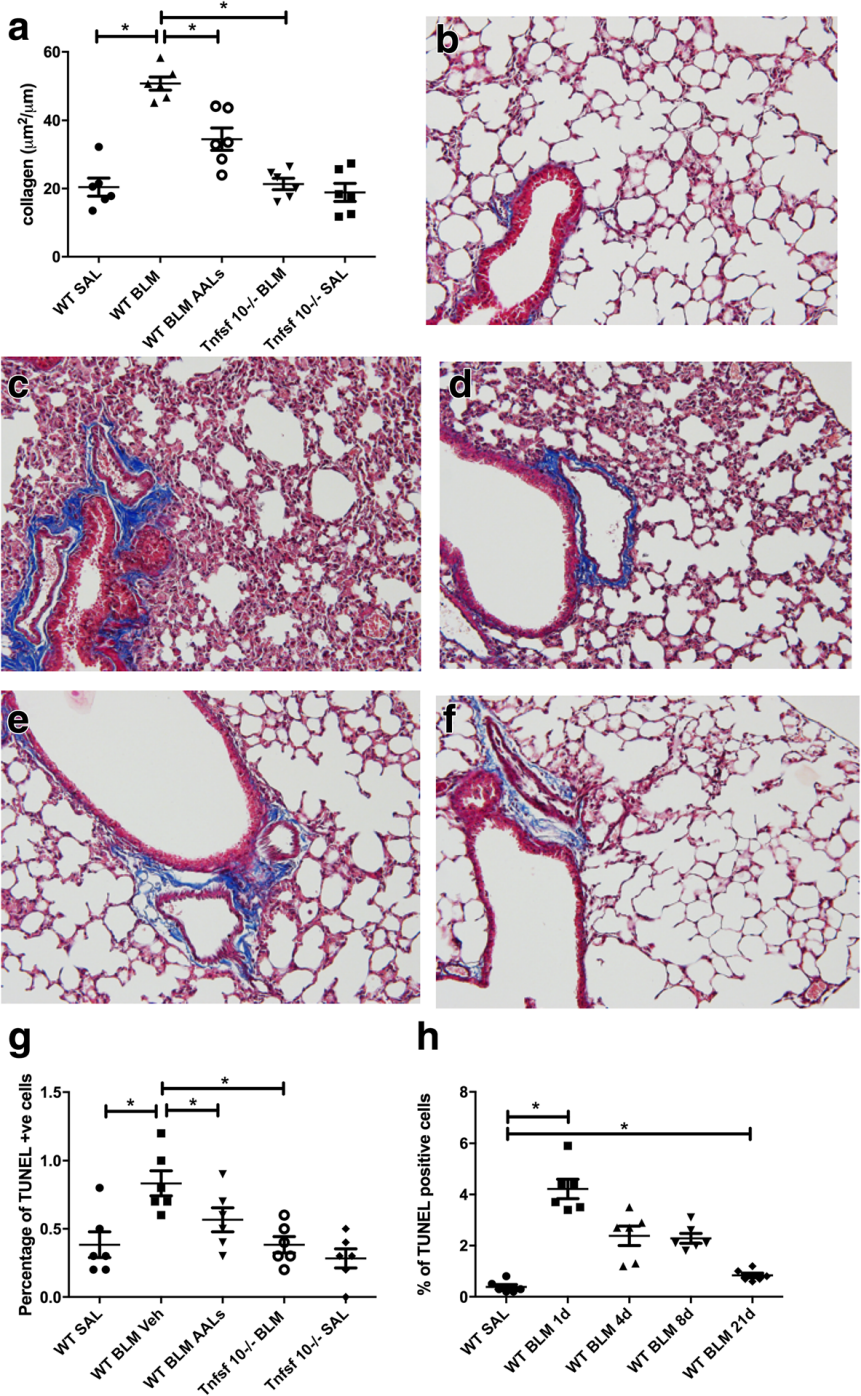
AAL(s) treated wild type mice and *Tnfsf10*^−/−^ mice were protected from bleomycin-induced collagen deposition and apoptosis. Collagen deposition in Masson’s trichrome stained lung sections (**a**). Images at 200x magnification of representative sections from Masson’s trichrome for (**b**) Wild Type saline and (**c**) bleomycin treated (**d**) wildtype bleomycin treated with AAL(s), (**e**) TRAIL−/− bleomycin treated and (**f**) TRAIL −/− saline. Apoptosis in terms of the percentage of TUNEL positive cells in lung sections (**g**), which peaked at one day post bleomycin but was still significantly elevated after day 21(**h**). *n* = 6, * *p* < 0.05

We then investigated the levels of collagen deposition and apoptosis. Histological investigation of Masson’s trichrome stained transverse lung sections demonstrated that bleomycin induced increases in collagen deposition around the airways compared to saline treated controls (Fig. 5a). AAL(s) treated WT mice and *Tnfsf10*^*−/−*^ mice had reduced or inhibited collagen deposition, respectively. TUNEL staining also revealed that bleomycin induced increases in the percentage of TUNEL positive cells that peaked after one day but was still elevated after 21 days (Fig. 5b, c). AAL(s) treated WT mice and *Tnfsf10*^*−/−*^ mice have reduced levels of TUNEL positive staining.

### Proliferation and release of Mid1 and collagen-α2(I) mRNA were increased in mouse lung fibroblasts treated with rTRAIL

**Fig. 6:**
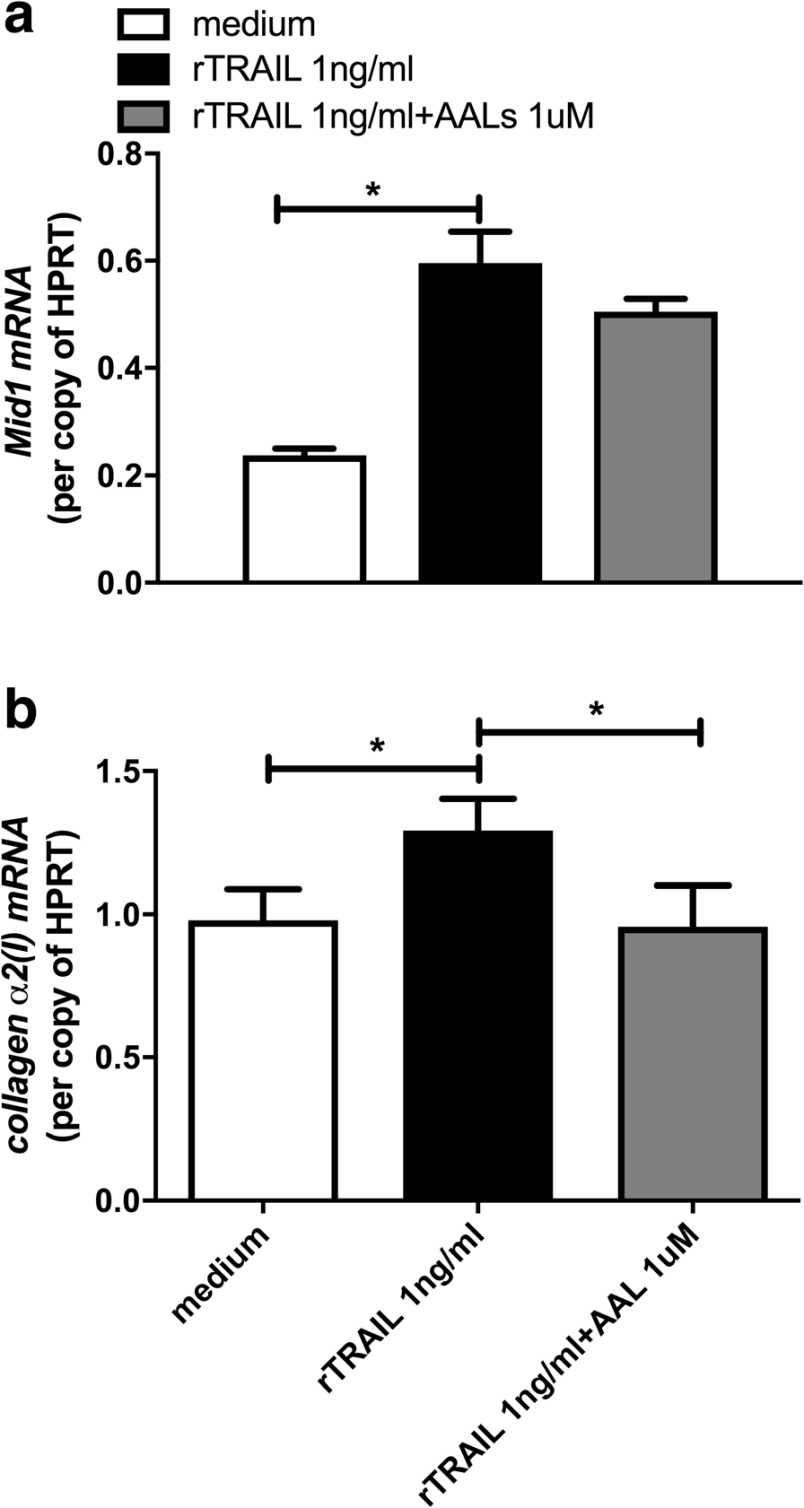
rTRAIL stimulated Mid1 and collagen- α2(I) production in isolated primary mouse fibroblasts. Mid1 (**a**) and collagen-α2(I) (**b**) expression per copy of HPRT in cultured fibroblasts. *n* = 3 independent experiments * *p* < 0.05

Next, we isolated fibroblasts from mouse lungs and cultured them with increasing concentrations of rTRAIL in-vitro. Over 24 h we found that proliferation of fibroblasts increased in the presence of TRAIL in a dose-dependent manner peaking at a dose of 1 ng/ml (data not shown). This dose was sufficient to induce the expression of Mid1 and collagen-α2(I) in fibroblast cultures, while collagen expression but not Mid1 was inhibited by addition of AAL(s) (Fig. 6 a, b).

## Discussion

We have previously demonstrated that TRAIL signalling through the ubiquitin ligase Mid1 and dysregulation of PP2A activity plays a key role in lung fibrosis in house dust mite and ovalbumin-induced allergic airways disease models [17, 22, 32]. The pro-inflammatory TRAIL pathway also is required for fibrosis in *Aspergillus fumigatus* and ovalbumin*-*induced models of eosinophilic oesophagitis and cigarette smoke induced models of COPD [16, 28, 33].

Here we show that TRAIL and MID1 are associated with pathogenesis and remodelling in IPF. TRAIL in patient serum and MID1 in the lungs of patients with active IPF are both upregulated (Fig. 2a, b). MID1 protein had an inverse correlation to lung function DLco in these IPF patients (Fig. 2e) while levels of PP2A activity, the effector of the pathway, were directly correlated (Fig. 2f). McGrath et al.*,* showed lower levels of TRAIL in the serum of IPF patients, though they did not examine signalling downstream of TRAIL [12] . More recent studies have shown elevated levels of TRAIL in airway epithelial cells isolated from active regions within IPF lungs [34] which is in line with our findings of increased MID1 and decreased PP2A activity in lung biopsies. Furthermore, post-hoc analysis of Schiller et al.’s recent proteomic profile including eleven IPF patient biopsies vs three healthy controls demonstrates MID1 to be significantly upregulated [35] (supplementary table s1 *p* = 0.012).

Our study expands on the previous studies by showing MID1 protein upregulation in IPF lung tissue and characterising the downstream signalling pathways affected by it in the form of reduced phosphatase activity of PP2A. Of particular interest, is the association of PP2A activity levels with a decline in DLco. This suggests that there may be a causal relationship to IPF pathogenesis supporting potential modulation of activity or the negative regulator E3 ubiquitin ligase MID1 as a therapeutic target. In support of this are our observations that AAL(s) treated WT mice and *Tnfsf10*^*−/−*^ mice were protected from bleomycin-induced fibrosis. However, these data contrast with the previous McGrath et al., study that demonstrated an increase in bleomycin-induced fibrosis in *Tnfsf10*^*−/−*^ mice [12]. McGrath et al., used female mice on a C57BL/6 background while we used male mice on a BALB/c background suggesting the mouse strain or gender may exert a significant influence on the bleomycin model in the absence of TRAIL. This is of interest as IPF is biased towards males though the reason for this is unknown [36]. We have also found that female *Tnfsf10*^*−/−*^ mice on a BALB/c background to spontaneously develop lung fibrosis associated with the small airways later in life [28], though these changes themselves may be due to alterations in the microbiome with repeated unfiltered room air exposures in the models used [28, 37]. Together, this suggests that there may be gender specific roles for either inflammatory or apoptotic TRAIL signalling that also play a role in lung fibrosis and is worthy of further investigation. It also highlights that experimental data from murine models need to be interpreted in the context of disease observed in patients where TRAIL and its downstream signalling pathway is augmented.

The complexity of the gender and strain influences observed in in-vivo models lead us to follow a more reductionist approach and conduct in-vitro cell culture experiments. This approach was previously employed to demonstrate that MID1-PP2A signalling is induced in primary human epithelial cells in the presence of recombinant TRAIL [22]. In fibrotic lung models TRAIL has been shown to be broadly expressed by multiple cells types including epithelial cells, endothelial cells, fibroblasts and alveolar macrophages [12, 17, 22, 28, 38]. Here we have demonstrated in cultured primary fibroblasts recombinant TRAIL directly induced both proliferation and the production of collagen in a dose dependent manner (Fig. 6). Furthermore, AAL(s) inhibited collagen expression without affecting Mid1 expression in-vitro. In contrast AAL(s) treated WT mice showed reduced Mid1 expression. This supports our proposal that PP2A is downstream of Mid1 on a cellular level while the effect of PP2A activation on Mid1 expression in-vivo occurs because of a blunted inflammatory and pro-fibrotic lung response involving a complex interaction between multiple cell types that cannot be replicated in isolated cells in-vitro. Thus, this combination of ex-vivo and in-vivo studies add further mechanistic support to a role for Mid1 in the promotion of fibrosis.

The key role for MID1 in fibrosis suggests this pathway may be suitable as a therapeutic target. The FTY720 analogue Fingolimod is undergoing phase 2 and phase 3 clinical trials involving patients with relapsing and relapsing-remittent multiple sclerosis [39], and was generally well tolerated at the approved dose [40]. However, FTY720 in its phosphorylated form is also a sphingosine 1-phospate receptor modulator, which inhibits lymphocyte migration and results in lymphopenia. Therefore, non-phosphorylatable FTY720 analogues such as AAL(s) and small molecules targeting MID1 may be more suitable for clinical trials in IPF patients, should any be available in the future.

A limitation of our study was that the diagnosis of IPF in these participants was reliant on transbronchial biopsies which is not in accordance with current clinical guidelines. To address the unknown specificity of our approach we excluded the possibility of IPF participants being incorrectly classified as controls by using control tissues distal to resected lung cancer in patients with no clinical evidence of IPF or other fibrotic lung disease. In regard to the unknown specificity of using transbronchial biopsies for the diagnosis of IPF our approach could have resulted -if anything- in a smaller difference in MID1 between IPF patients and controls in our study. However, despite this limitation, we found a significant difference in serum TRAIL, tissue MID1 and a trend in PP2A between groups which is suggestive of consistent and robust data. Though further studies utilising tissue from a population with clinical diagnosis according to the most recent Fleischner Society White Paper [41] could now be warranted to precisely identify which patients would most benefit from novel therapeutics targeting MID1.

## Conclusions

TRAIL signalling through MID1 deactivates PP2A and promotes fibrosis with corresponding lung-function decline. This may provide novel therapeutic targets for IPF.
